# Recurrent Reversible Cerebral Vasoconstriction Syndrome: A Report of Two Cases

**DOI:** 10.7759/cureus.42992

**Published:** 2023-08-05

**Authors:** Pooja A Patel, Emma Sanborn, Ryna Then, Dena M Williams

**Affiliations:** 1 Internal Medicine, Rutgers University New Jersey Medical School, Newark, USA; 2 Neurology, University of Rochester Medical Center, Rochester, USA; 3 Neurology, Cooper Medical School of Rowan University, Camden, USA; 4 Neurology, The University of North Carolina at Chapel Hill, Chapel Hill, USA

**Keywords:** neuro-vascular, reversible cerebral vasoconstriction syndrome (rcvs), ischemic cerebrovascular disease, intracerebral hemorrhage, headache, vascular neurology, stroke

## Abstract

Reversible cerebral vasoconstriction syndrome (RCVS) is a rare neurological condition that classically presents with recurrent, thunderclap headaches and radiographic findings of multifocal narrowing of cerebral vasculature. Complications of RCVS may include ischemic or hemorrhagic strokes. Sympathomimetic agents including cannabinoids have been associated as precipitants in many cases. RCVS is classically considered to be reversible, although cases of recurrent RCVS have been described in the literature. In this report, we describe two cases of recurrent RCVS, which were precipitated by recurrent exposures to inciting agents. The first patient was found to have a history of repeated exposure to tetrahydrocannabinol (THC) and suffered from recurrent multifocal ischemic strokes with evidence of persistent multifocal narrowing of cerebral vasculature by cerebral arteriography. The second case describes a patient with a history of use of ashwagandha, medical marijuana, and serotonin-norepinephrine reuptake inhibitors (SNRIs) who experienced multiple intracranial hemorrhages with radiographic evidence of multifocal narrowing of cerebral vessels as well.

## Introduction

Reversible cerebral vasoconstriction syndrome (RCVS) is a rare neurological condition that is characterized by recurrent, thunderclap headaches with imaging findings of multifocal vasoconstriction of cerebral arteries [1]. RCVS can cause permanent neurologic deficits by means of ischemic stroke and intracerebral hemorrhage [2,3]. The two major pathophysiological mechanisms of RCVS currently under investigation are endothelial dysfunction and alterations in cerebral vascular tone [1,3]. Sympathomimetic agents such as cannabinoids, selective serotonin reuptake inhibitors (SSRIs), and nasal decongestants have been identified as possible precipitants in many cases, as has the postpartum state [1]. RCVS is typically a transient phenomenon with a generally benign clinical course. In most patients, there is both clinical and radiographic recovery noted within three months [4]. However, in some patients, severe complications may develop including ischemic stroke, intracerebral hemorrhage, and subarachnoid hemorrhage [1]. In two large cohorts, up to 6% of patients were reported to experience a recurrence of RCVS [5,6]. The diagnosis of RCVS requires a high clinical suspicion with recognition of the typical signs and symptoms of the condition, commonly recognized precipitating agents, and common radiographic features. Management of RCVS involves avoidance of the precise precipitating agent once identified [7]. In our report, we present two atypical cases of recurrent RCVS associated with recurrent exposures to precipitating agents and profound neurological complications.

## Case presentation

Case 1

**Figure 1:**
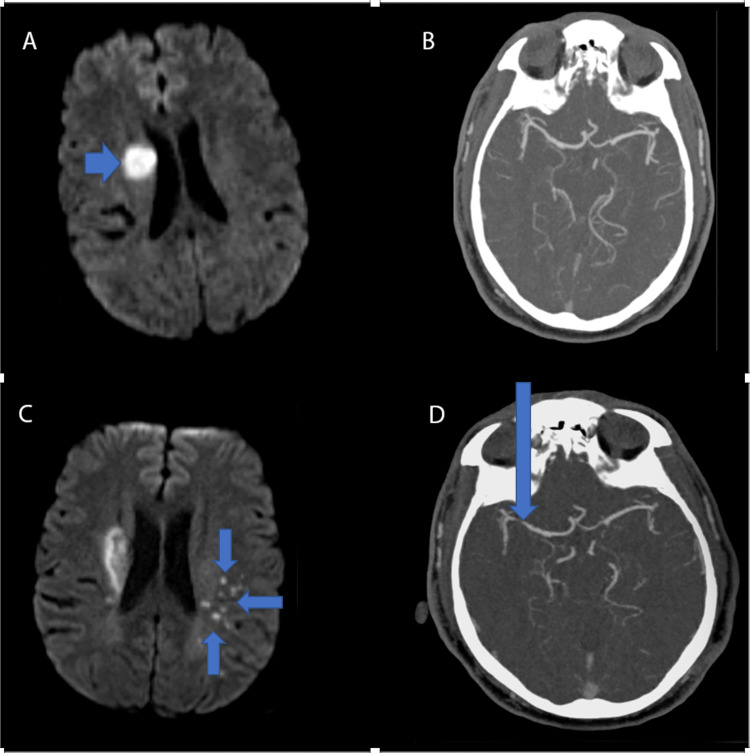
Neuroimaging findings for Case 1. (A) MRI Brain without contrast demonstrating right corona radiate ischemic stroke; (B) Initial CTA Head and Neck with contrast demonstrating normal findings; (C) MRI Brain without contrast demonstrating new, multiple, punctate infarcts in the left frontoparietal area; (D) CTA Head and Neck with contrast revealing new stenosis of the distal right M1. MRI: magnetic resonance imaging; CTA: computed tomography angiography

A 52-year-old male with a past medical history of uncontrolled hypertension presented with new-onset severe, thunderclap headache, dysarthria, and left-sided facial droop. The patient admitted to a history of substance use, in the form of tetrahydrocannabinol (THC). Urine drug screen (UDS) tested positive for THC. Vital signs on initial admission were notable for elevated blood pressure with average systolic blood pressure in the 200s. Neurologic examination revealed mild dysarthria and mild left facial droop involving both the upper and lower face with predominant lower facial weakness compared to upper facial weakness. An uneven distribution can often be seen in central nervous system etiologies. Magnetic resonance imaging (MRI) of the brain without contrast demonstrated a right corona radiata (CR) infarct (Figure 1a). Initial computed tomography angiography (CTA) of head and neck with contrast was unremarkable without any apparent vessel stenosis or narrowing (Figure 1b). The patient was outside the window for any acute intervention. A routine stroke workup was completed, including a lipid panel with low-density lipoprotein (LDL) of 97 and glycosylated hemoglobin of 5.9%. In addition, a transthoracic echocardiogram (TTE) was performed and demonstrated a normal ejection fraction (EF) of 50%, normal left atrial size, and positive identification of patent foramen ovale (PFO). Lower extremity duplex ultrasound (US) was negative for deep venous thrombosis (DVT). The patient was started on aspirin 81 mg and atorvastatin 80 mg for secondary stroke prevention.

During this hospitalization, the patient’s clinical course worsened during hospital days 2-3 with the development of new-onset left arm and leg weakness. On examination, he exhibited 2/5 muscle strength in the left upper extremity and 3/5 muscle strength in the left lower extremity. Repeat MRI of the brain without contrast revealed enlargement of the previously seen right CR infarct consistent with expected evolution as can be seen with lacunar strokes. His average systolic blood pressure improved to the 150s over several days during his hospitalization. He was started on two maintenance antihypertensive medications, lisinopril 10 mg and hydrochlorothiazide 12.5 mg, for optimal blood pressure control. After his exam worsened, clopidogrel 75 mg was added to his anti-platelet regimen for a total duration of 21 days in addition to aspirin 81 mg. The authors recognize that the patient care at this point should have been in accordance with the 2013 Clopidogrel in High-Risk patients with Acute Nondisabling Cerebrovascular Events (CHANCE) protocol since the patient had a National Institutes of Health Stroke Scale (NIHSS) score of >3 when clopidogrel was added and the patient was several days out from initial symptoms. Additionally, the patient was eligible for dual antiplatelet therapy (DAPT) on initial presentation. The authors cared for the patient at a later time frame and recognize this missed opportunity. After eight days of inpatient hospitalization and improvement in clinical symptoms, the patient was discharged to inpatient rehabilitation with the plan to undergo outpatient cardiac monitoring to evaluate for arrhythmias. In addition, the patient was extensively counseled on the importance of abstaining from tetrahydrocannabinol use, given it is a known risk factor for stroke. At this time, RCVS was also considered given the report of thunderclap headache on admission and THC use; however, vessel imaging did not support the diagnosis. The presumed etiology of the patient's stroke was small vessel disease in the setting of uncontrolled hypertension. 

Less than 30 days later, the patient returned to the hospital with new mild expressive aphasia and right-sided facial droop. The patient endorsed that he was still experiencing headaches, which he continued to describe as thunderclap in nature. MRI of the brain without contrast revealed multiple acute punctate infarcts in the left frontoparietal area (Figure 1c). CTA Head and Neck with contrast demonstrated findings concerning for new stenosis of the distal right M1 (Figure 1d), which was not seen on the initial CTA. UDS resulted positive for THC again. Digital subtraction angiography (DSA) was done and revealed multifocal narrowing and beaded appearance of the intracranial circulation, greater on the right than the left. RCVS was the leading differential at this time given the patient’s reported thunderclap headaches, cerebral vessel imaging findings of multifocal narrowing and progression of intracranial vessels compared to prior vessel imaging, and persistent use of THC. Given the beaded appearance noted on DSA, the patient underwent a lumbar puncture to assess for the possibility of central nervous system vasculitis. Cerebrospinal fluid studies obtained from the lumbar puncture were within normal limits. Following the clinical improvement of neurological symptoms, the patient was started on nimodipine for symptomatic treatment of headaches and prevention of further vasospasm of cerebral arteries. Upon discharge, he was extensively counseled on medication compliance for outpatient nimodipine, recommended to abstain from THC use, and advised to perform a repeat CTA Head and Neck with contrast outpatient in three months to further assess intracranial vasculature.

Ten days later, he again returned with altered mentation and unsteady gait. MRI brain without contrast revealed new infarcts in the right middle cerebral artery (MCA) distribution, which were not identified in previous neuroimaging. CTA Head and Neck with contrast revealed progression of the irregularities noted at the second admission, particularly in the right MCA branches. He was again admitted for further workup and management of his stroke. UDS was negative for THC. Lumbar puncture and complete serum rheumatologic and hypercoagulable workup were repeated and unrevealing. At this time, primary central nervous system angiitis (PACNS) was considered on the differential diagnosis, and the patient was started on pulse dose steroids of 1 g daily of intravenous methylprednisolone for five days. Rheumatology and Ophthalmology were consulted and found no concern for primary or secondary central nervous system vasculitis. As there was a lower concern for PACNS and the patient had no significant improvement on pulse dose steroids, pulse dose steroids were discontinued after three days of treatment. The neurology team led a multi-disciplinary discussion and review of the patient's case with vascular neurologists, neurointerventionalists, and leading disease-specific experts who have published many papers on RCVS (Personal communication; Singal AB, Harvard Medical School, Boston, Massachusetts, and Calabrese L, Cleveland Clinic, Cleveland, Ohio). The patient's recurrent presentations were most likely secondary to RCVS and the repeated exposure of THC may have led to a more progressive vasculopathy that was possibly irreversible. Extensive workup was unrevealing for other entities such as a primary or secondary CNS vasculitis and the time course was too rapid and acute to be caused by atherosclerosis. During this hospitalization, the patient was transitioned from nimodipine to verapamil for treatment of RCVS and optimization of blood pressure as this regimen was felt to be a better long-term medication. The patient was hospitalized for 30 days after which time he was discharged home on a regimen including aspirin, atorvastatin, and verapamil.

Three months after discharge, the patient underwent repeat DSA, which revealed persistent multifocal stenosis throughout the intracranial circulation with minimal progression in the distal right MCA. 

Case 2

A 47-year-old female with past medical history of hypertension, Hashimoto’s thyroiditis, and bipolar disorder presented as a transfer from an outside hospital with symptoms of headaches described as thunderclap in nature and neck pain for approximately two weeks. On neurological examination, there were no focal neurological deficits appreciated. The patient admitted to the use of medical marijuana and ashwagandha, an Indian herb often considered to have neuroprotective effects and reported to increase thyroid hormone levels [8]. Stroke protocol CT Head without contrast and MRI Brain without contrast were obtained at an outside hospital prior to transfer and demonstrated a small volume right frontoparietal cortical subarachnoid hemorrhage (SAH). CTA Head and Neck with contrast from the outside hospital was negative for any evidence of aneurysm. The location of SAH in the cortex was not indicative of an aneurysmal SAH. DSA was performed given the patient's spontaneous, non-aneurysmal SAH to evaluate for an etiology and revealed a beaded appearance of the right MCA vessel and its branches, which correlated with the location of the SAH. UDS did not reveal evidence of use of common recreational substances. Rheumatological workup revealed mild elevation of erythrocyte sedimentation rate (ESR) at a value of 34 mm/hour (normal range 0-27, when corrected for age). Given reported recurrent thunderclap headaches, small volume SAH, and appearance of the right MCA vessels with notable triggers of ashwagandha and marijuana use, RCVS was deemed the likely cause of this patient's spontaneous SAH. The team intended to start a calcium channel blocker for symptomatic treatment of headaches likely secondary to RCVS; however, the patient left against medical advice prior to being able to do so.

**Figure 2:**
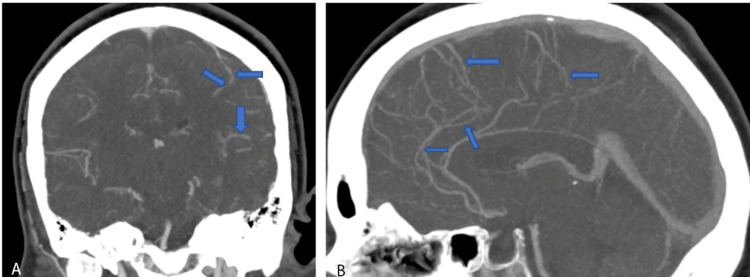
CTA of the head and neck in Case 2 with contrast demonstrating multifocal stenosis of intracranial arteries. CTA: computed tomography angiography

The patient returned within two weeks with worsening thunderclap headaches and blurry vision. Neurologic examination was again unremarkable for any focal neurological deficits. CT Head without contrast was unrevealing for any acute intracranial pathology. CTA Head and Neck with contrast demonstrated multifocal stenosis of intracranial arteries, right greater than left (Figure 2). Given persistent concern for RCVS, the patient was treated with verapamil for symptomatic relief. Following improvement in her symptoms, she was discharged home with the plan for continuation of verapamil and follow-up with neurology outpatient with repeat imaging. She was also extensively counseled to avoid ashwagandha and other stimulants to avoid precipitating RCVS.

Two months later, the patient followed up with outpatient neurology clinic and noted resolution of her headaches. Follow-up CTA Head and Neck with contrast demonstrated resolution of multifocal stenosis of cerebral arteries, which is typically seen with RCVS. The patient was advised to continue verapamil as prophylaxis at the discretion of the treating physician and maintain regular neurology outpatient follow-up visits.

**Figure 3:**
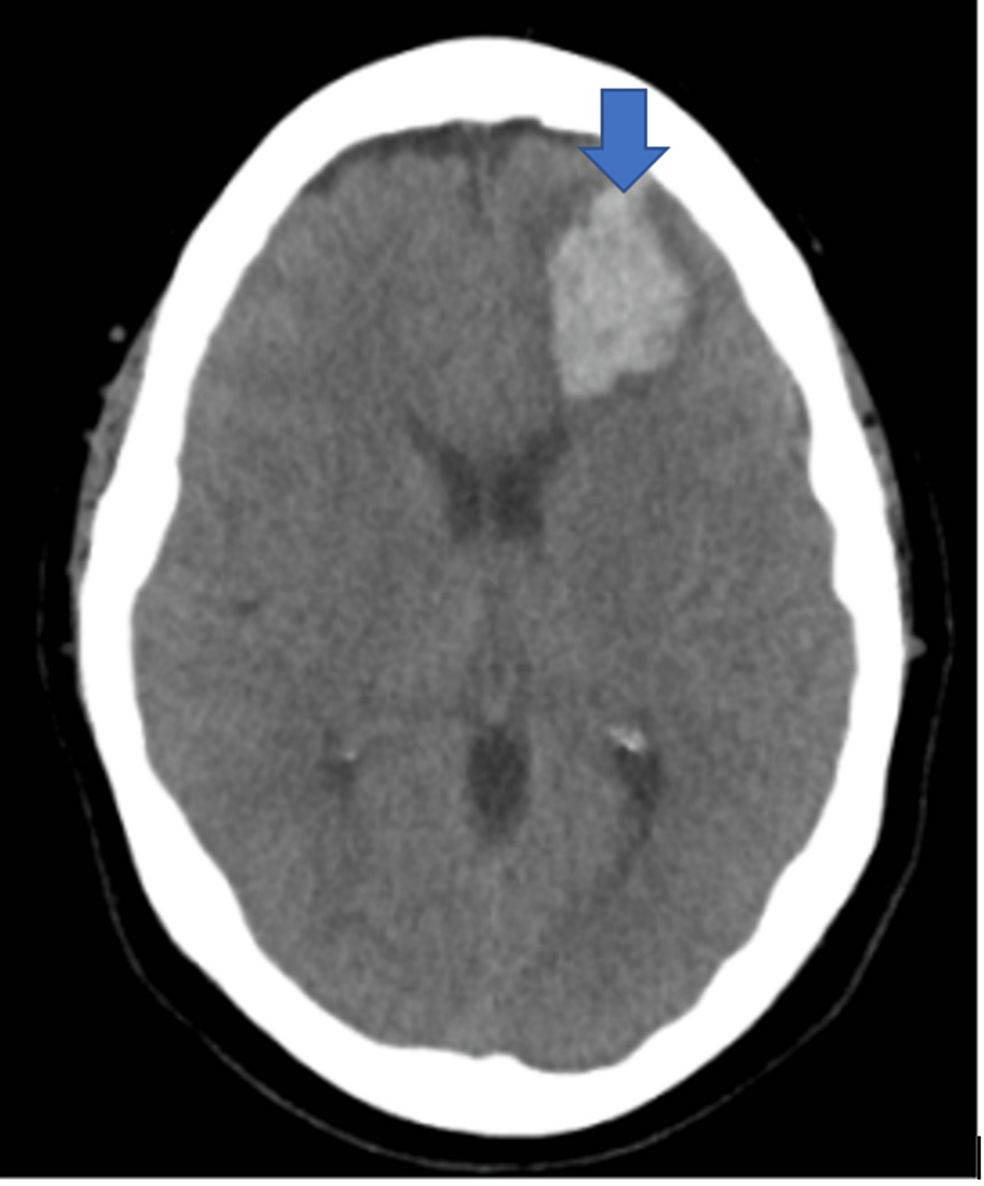
CT Head without contrast in Case 2 demonstrating new left frontal cortical intraparenchymal hemorrhage.

**Figure 4:**
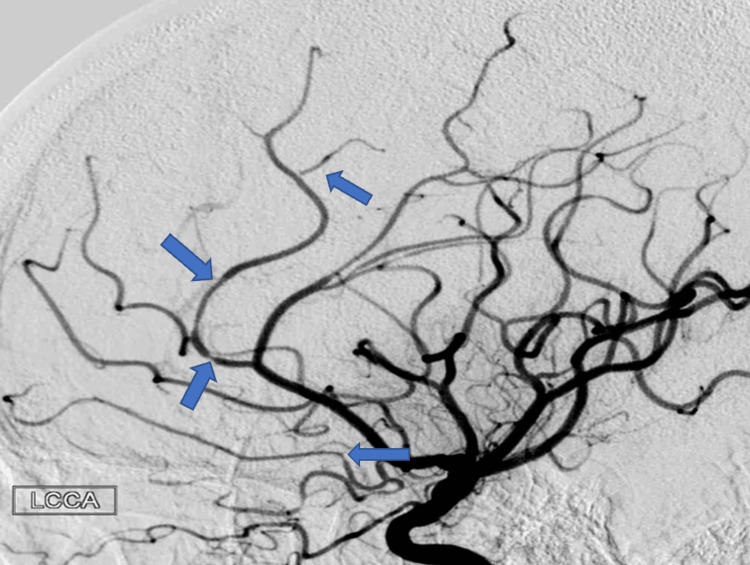
Digital subtraction angiogram of left internal carotid arteries in Case 2 following the patient’s first occurrence of IPH, demonstrating multifocal stenosis of the anterior intracranial circulation. IPH: intraparenchymal hemorrhage

**Figure 5:**
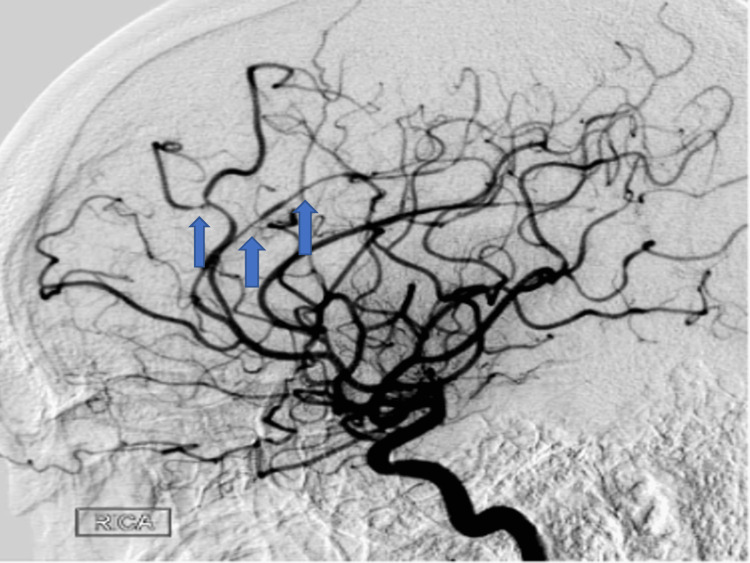
Digital subtraction angiogram of right internal carotid arteries in Case 2 following the patient’s first occurrence of IPH, demonstrating multifocal stenosis of the anterior intracranial circulation. IPH: intraparenchymal hemorrhage

A year after her last outpatient appointment, she presented to the hospital with recurrent episodic thunderclap headaches with associated nausea, vomiting, and photosensitivity. Her neurologic examination was notable for aphasia without notable weakness. In the interim, she had established care with another neurologist who was closer to her home, who had discontinued verapamil and started the patient on venlafaxine for treatment of headaches and mood. CT Head without contrast revealed bilateral SAHs and a left frontal intraparenchymal hemorrhage, approximately 9.4 cc by ABC/2 formula (Figure 3). CTA Head and Neck with contrast again demonstrated multifocal stenosis of intracranial arteries. She underwent a second DSA, which demonstrated multifocal stenosis of intracranial arteries bilaterally (Figures 4-5). Angiogram found diffuse irregularities primarily affecting bilateral anterior cerebral arteries and, to a lesser extent, the bilateral middle cerebral and right superior cerebellar arteries. The patient also underwent brain biopsy with hematoma evacuation. Rheumatological workup was repeated and unremarkable. Lumbar puncture was performed and notable for mildly elevated glucose of 85 (normal range: 50-75 mg/dL) and mildly elevated protein at 46 (normal range: 15-45 mg/dL). Due to concern for PACNS as per discretion of treating physician at that time given the recurrence of symptoms and vascular irregularities, she was administered one dose of high-dose IV methylprednisolone, but this was discontinued when brain biopsy was planned. The biopsied temporal artery, dura, and brain showed no evidence of vasculitis. In the absence of any evidence for an ongoing inflammatory process, verapamil was restarted for symptomatic treatment of suspected recurrent RCVS. It was felt that RCVS was likely precipitated to exposure to SNRI. Venlafaxine was discontinued as this is a known precipitant of RCVS. After clinical improvement, the patient was discharged home and advised to continue verapamil, follow up with vascular neurology outpatient clinic, and undergo follow-up repeat vessel imaging.

**Figure 6:**
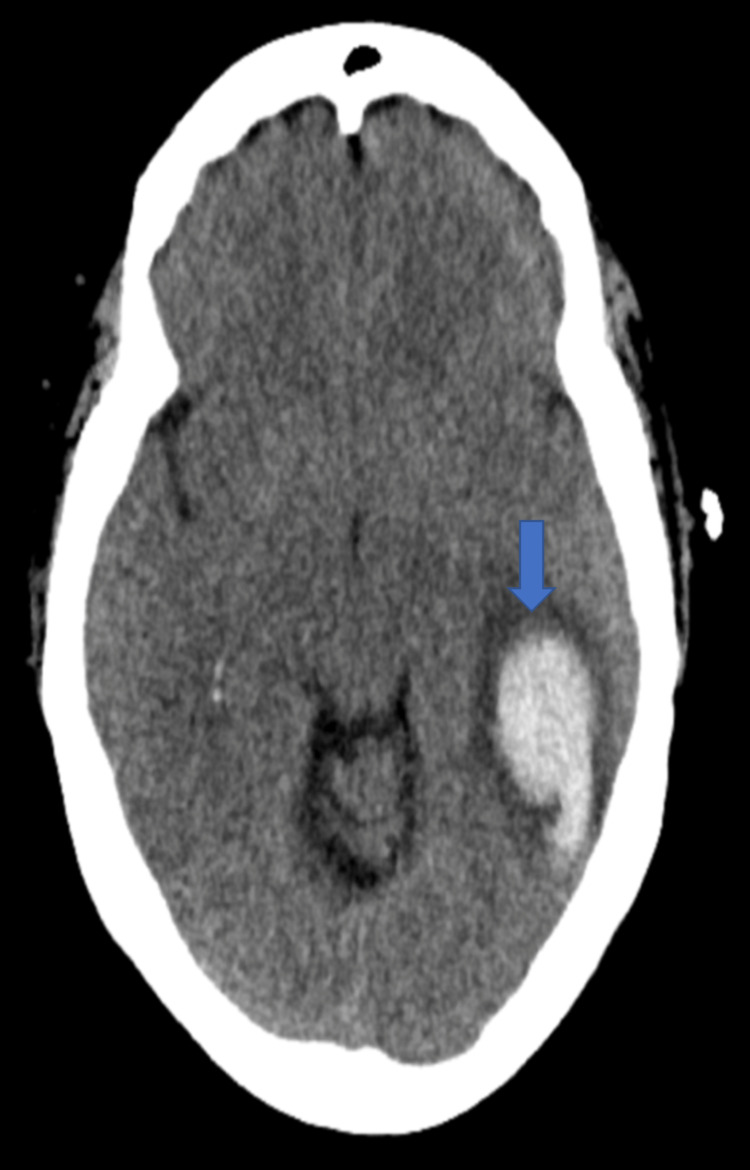
CT Head without contrast in Case 2 demonstrating acute left temporal intraparenchymal hemorrhage.

Unfortunately, she returned to the hospital one week later with new onset right facial weakness and receptive aphasia. CT Head without contrast now revealed an acute left temporal lobe hemorrhage approximately 5.5 cc by ABC/2 formula (Figure 6). CTA Head and Neck with contrast demonstrated increased multifocal irregularities compared to prior imaging studies. Patient's home dose of verapamil was increased. The patient improved clinically and at the time of discharge, she had residual receptive aphasia and trace right facial weakness. There was suspicion that the patient was using medical marijuana for symptomatic relief of headache pain as per collateral obtained from patient's family. At the time of discharge, the patient was extensively counseled to avoid all possible precipitants of RCVS. 

**Figure 7:**
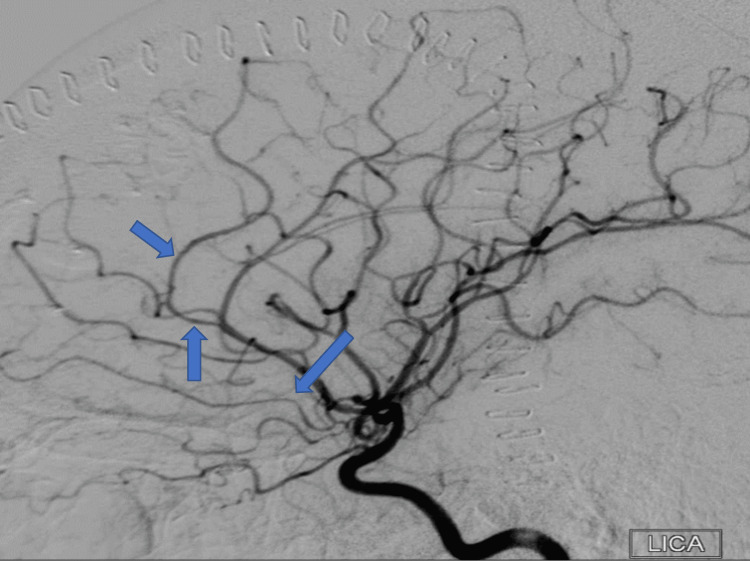
Digital subtraction of the left internal carotid arteries in Case 2 demonstrating improvement in multifocal stenosis seen on angiogram two weeks prior.

**Figure 8:**
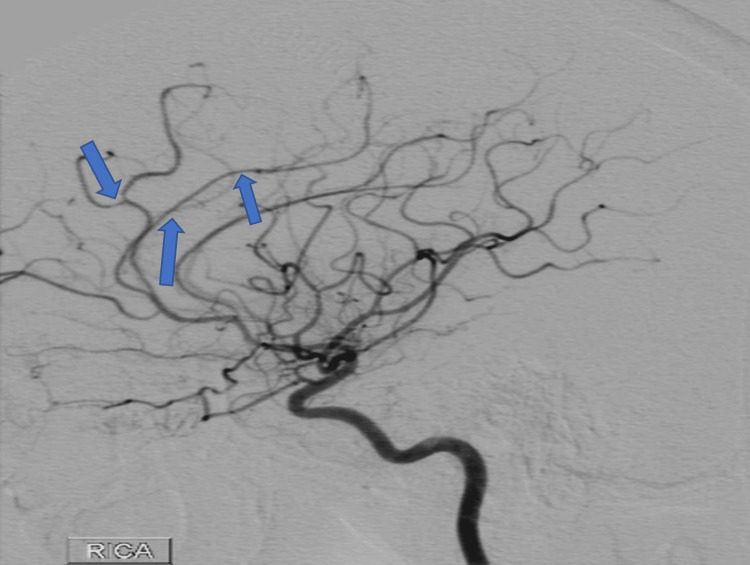
Digital subtraction of the right internal carotid arteries in Case 2 demonstrating improvement in multifocal stenosis seen on angiogram two weeks prior.

Two weeks later, the patient underwent a repeat DSA, which showed interim improvement in multifocal stenosis with near resolution of the stenoses seen in the MCAs (Figures 7-8). During this study, a fistula between the right parietal middle meningeal artery (MMA) and the distal superior sagittal sinus was noted. Approximately four months later, the patient underwent onyx embolization of the MMA-sagittal sinus fistula. At the time of the writing of this article, the patient has not had any further recurrence of vessel irregularities, although she had been admitted for a brief hospital stays with complaint of headaches. Resolution of cerebral vessel irregularities has been confirmed on repeat DSA performed for arteriovenous fistula embolization.

## Discussion

RCVS is a rare neurological condition that classically presents with severe headaches and vasoconstriction of cerebral vessels on cerebral angiography. It has a generally benign clinical course with the resolution of clinical symptoms and radiographic changes after the withdrawal of inciting agents within three months. The mainstay treatment of RCVS is avoidance of inciting triggers and calcium channel blockers. Calcium channel blockers are commonly used for symptomatic relief of headaches and for the prevention of cerebral vasoconstriction. While evidence suggests that calcium channel blockers are beneficial for headache relief, it is less clear if they truly prevent vasoconstriction and thus, they may not have a curative effect [2,6]. Less commonly, the condition can lead to long-term disability and devastating effects if it leads to ischemic or hemorrhagic strokes [9,10].

Our cases highlight the increased morbidity that can result from recurrent episodes of RCVS as both patients suffered from complications of RCVS. These recurrences were presumed to be a result of repeated exposures to known precipitating agents. In the first case, the patient experienced recurrent ischemic infarcts in the setting of continued use of THC, which ultimately resulted in persistent multifocal vascular irregularities. The authors of this article presume RCVS as the etiology of this clinical presentation and radiographic findings. There was a lack of evidence to suggest inflammatory processes that could cause vasculitides and the time course was too rapid to be consistent with ischemic infarcts from atherosclerotic disease. Similarly, the patient in the second case experienced multiple intracranial hemorrhages as a result of exposure to various agents including marijuana, SNRIs, and ashwagandha. While she had reversibility and improvement of radiographic changes of cerebral vasoconstriction, the recurrent exposures to the presumed precipitating agents and multiple hemorrhagic infarcts led to permanent long-term deficits.

While RCVS is a rare neurological condition, it is imperative for clinicians to have an understanding of this disease process and the many precipitating agents that may trigger it. Likewise, it is essential to provide extensive education to patients regarding potential precipitating agents and the importance of abstinence from these common precipitants to prevent recurrence and disability from the condition. To our knowledge, there is no relevant data on recurrent RCVS with repeated exposure and the devastating long-term consequences that may result. 

## Conclusions

The two cases featured in this report highlight the potentially devastating and irreversible effects that patients with RCVS may experience due to recurrent exposures to precipitating sympathomimetic agents. Physicians should be aware of the potentially life-threatening complications when caring for patients with RCVS. Recurrent episodes of RCVS can impair patients’ functional abilities and thus result in long-term morbidity. There is still much that remains unknown about the condition and complications that may result from it. This case report highlights the need for thorough patient education in regard to avoidance of potential precipitants and long-term follow-up to prevent the adverse outcomes that can be associated with this condition.
